# Signal Processing Platform for Long-Range Multi-Spectral Electro-Optical Systems

**DOI:** 10.3390/s22031294

**Published:** 2022-02-08

**Authors:** Nikola Latinović, Ilija Popadić, Branko Tomić, Aleksandar Simić, Petar Milanović, Srećko Nijemčević, Miroslav Perić, Mladen Veinović

**Affiliations:** 1 Department for Postgraduate Studies, Singidunum University, Danijelova 32, 11000 Belgrade, Serbia; mveinovic@singidunum.ac.rs; 2 Vlatacom Institute of High Technologies, Milutina Milankovica 5, 11070 Belgrade, Serbia; ilija.popadic@vlatacom.com (I.P.); branko@vlatacom.com (B.T.); aleksandar@vlatacom.com (A.S.); petar.milanovic@vlatacom.com (P.M.); srecko@vlatacom.com (S.N.); miroslav.peric@vlatacom.com (M.P.)

**Keywords:** multi sensor electro-optical systems, video processing platform, smart sensor

## Abstract

In this paper, we present a hardware and software platform for signal processing (SPP) in long-range, multi-spectral, electro-optical systems (MSEOS). Such systems integrate various cameras such as lowlight color, medium or long-wave-infrared thermal and short-wave-infrared cameras together with other sensors such as laser range finders, radars, GPS receivers, etc. on rotational pan-tilt positioner platforms. An SPP is designed with the main goal to control all components of an MSEOS and execute complex signal processing algorithms such as video stabilization, artificial intelligence-based target detection, target tracking, video enhancement, target illumination, multi-sensory image fusion, etc. Such algorithms might be very computationally demanding, so an SPP enables them to run by splitting processing tasks between a field-programmable gate array (FPGA) unit, a multicore microprocessor (MCuP) and a graphic processing unit (GPU). Additionally, multiple SPPs can be linked together via an internal Gbps Ethernet-based network to balance the processing load. A detailed description of the SPP system and experimental results of workloads for typical algorithms on demonstrational MSEOS are given. Finally, we give remarks regarding upgrading SPPs as novel FPGAs, MCuPs and GPUs become available.

## 1. Introduction

Long-range surveillance electro-optical systems are designed for the observation of targets that are more than ten kilometers away from cameras. They typically utilize long focal length lances which have small fields of view (FOVs) of about one degree in the maximal zoom position [1,2]. In order to make its mission successful in various day/night and meteorological conditions, usually, different types of cameras are integrated in the system such as color, lowlight [1,3], short-wave infrared (SWIR) [4], medium-wave infrared (MWIR) or long-wave infrared (LWIR) cameras [5,6], together with other sensors such as laser range finders (LRFs) [7], gated laser illuminators (LIs) [8], etc. Thus, such a system is denoted as a multi-sensor electro-optical system (MSEOS). There is no general rule regarding the choice of the type of key components incorporated into the MSEOS because it significantly depends on the specific application. This is usually a subset of the following set:

- Pan-Tilt positioner with mounted mechanical housing;
- Thermal infrared camera with a suitable lens covering the infrared (MWIR) or long-wave infrared (LWIR) part of the spectrum;
- Short-wave infrared camera with a suitable lens for the short-wave infrared (SWIR) part of the spectrum;
- Lowlight camera with a suitable lens for the visible part of the spectrum;
- Digital magnetic compass (DMC);
- Global Positioning System (GPS);
- Laser range finder (LRF);
- Inertial Measurement Unit (IMU);
- Signal processing module with built-in algorithms and appropriate software.

An MSEOS’s sensors are installed on a pan-tilt positioner platform with the alignment of their optical axis. A pan-tilt positioner enables precise sensor block movement of about several tens of degrees in the elevation plane and continuous movement (N × 360°) in the azimuthal plane. The accuracy of p is selected to be less than half of an instantaneous field of view (pixel FOV) of all installed cameras, which is typically less than 1/1000 degree.

Traditionally, MSEOS power supply, raw video and control signal are transmitted through a slip ring [9] towards control and signal processing units. Nowadays, cameras predominantly have digital interfaces, so the transmission of raw video images would require very high bandwidth (e.g., 17.6 Gbps for 4 k, 60 frames per second and 12 bits per channel video), which could be only supported by a very expensive slip ring that has not only cooper contacts, but also fiber optics transmission. In order to overcome this problem, a signal processing unit should be integrated on the rotational part of a pan-tilt positioner platform together with other sensors. Bearing in mind complex signal processing tasks such as video stabilization [10], artificial-intelligence-based target detection, target tracking [11], video enhancement [12], target illumination or multisensory image fusion, a signal processing platform (SPP) should be a very powerful computer [13]. Additionally, MSEOS are installed outdoors, so SPPs should be capable of working in extended temperature ranges and harsh humidity conditions. All these key components can be considered as isolated complex subsystems based on advanced technology and directly affect the performance of an MSEOS system. It is very rare for a particular MSEOS system manufacturer to have technology with each of these subassemblies, so they are mostly purchased from third parties as original equipment modules (OEMs), and their integration is performed at the system level. The more resources manufacturers invest in the integration process, the more compact and smaller the MSEOS systems will be. Manufacturers most often approach the development of video signal processing modules. The challenge that arises in the development of such a module is to interact with all components of the system and meaningfully connect them into one functional assembly. To accomplish this, the module will host all software and all algorithms applied in the system appropriately. Some examples of modules that can be used for the described purpose are presented in [14,15,16]. The limitation of the mentioned solutions is mainly in the way of use, because they are intended for specific tasks and are not sufficiently modular for efficient integration into a compact MSEOS.

**Figure 1:**
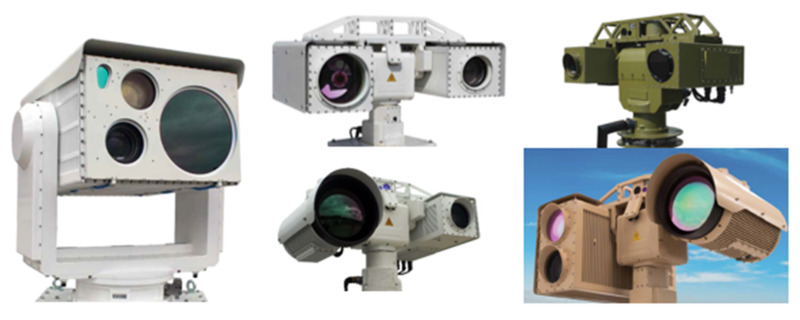
Some family members of VMSIS3 EO systems.

To overcome all the mentioned problems, we developed a new, highly integrated video signal processing platform in the form of a compact module, denoted as the Vlatacom Video Signal Processor (VVSP), which will expand the range of applications of advanced electro-optical systems. The module should enable the integration of key system components, enable managed communication and perform video signal processing using an efficient pipeline with minimized delay, while incorporating advanced video signal processing algorithms. Complex development includes the design of hardware, software, signal processing algorithms, network topology, as well as the method of integration into the system. Some early results about the VVSP module are presented in [17,18]. Today, the VVSP is a mature product integrated in multiple MSEOS, denoted as Vlatacom Multi-Sensor Imaging Systems, third generation (VMSIS3), which are shown in Figure 1.

This paper describes in detail all the key challenges that were addressed during the process of designing such a complex module. The architecture of the module is presented in detail, and special attention is paid to the approach of distributed data processing, as well as the method of module integration in the EO system. The validation of the quality of the VVSP module and the entire EO system was achieved through in-field application at the national and international level.

This introductory section is followed by a detailed description of the hardware, software, algorithm, integration and results. In Section 2, the hardware architecture, signal processing architecture, network architecture and system integration are described in detail. In Section 3, certain results regarding performances of the developed algorithms and the behavior of the VVSP module under different temperature ranges are presented. In Section 5, the results and presented methodology with knowledge adopted during the development of this project are discussed along with possible future upgrades of the system.

## 2. Description of VVSP Module

**Figure 2:**
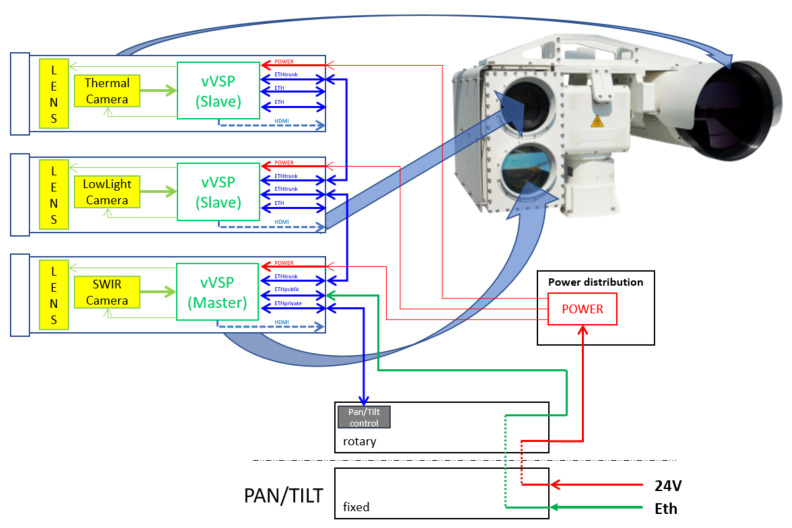
Distributed VMSIS3 architecture.

The VMSIS3 is designed to use the modularity feature of the VVSP module by applying it in a distributed architecture, thus enabling system scalability. The VVSP module is designed to have enough resources to process the video signals coming from a single camera. If a new camera is added to the system, new VVSP modules are added and connected in cascade with the previous one, so that there is no overload. An example of a distributed architecture of the VMSIS3 system is shown in Figure 2. In this way, the complete processing of the video signal and the application of appropriate algorithms is performed on a single VVSP module that is integrated in the housing together with the camera from which it receives the video signal.

The VVSP module was completely developed at the Vlatacom Institute and represents its intellectual property. Complex development includes the design of hardware, software, signal processing algorithms, network topology, as well as the method of integration into the system. All aspects of VVSP module development are described in this paper.

### 2.1. Hardware Architecture

It is clear that the VMSIS3 relies on the VVSP, so this module within the EO system is responsible for controlling all devices, the complete manipulation of the video signal, as well as for communication, both internally within the system and externally with the command control point. In order to accomplish demanding tasks, the VVSP engages state-of-the-art technologies. This is achieved by applying one application module and one FPGA module, each of which performs the tasks for which it is intended. The Nvidia Jetson TX2 [19] with a quad-core arm and 256 CUDA graphics cores was selected for the application module. On the other hand, the FPGA technology is integrated through the Xilinx KINTEX-7 [20] with 160 k logic inputs on the Enclustra KX2 module [21]. This form factor can be upgraded to Xilinx Zynq UltraScale+ MPSoC [22].

**Figure 3:**
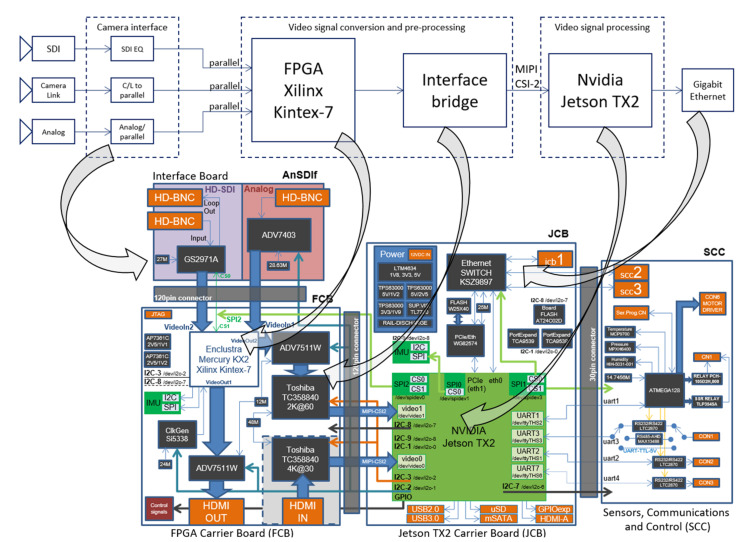
Video signal path (**top**) and VVSP module architecture (**bottom**).

Complete VVSP architecture based on the two abovementioned modules with a video signal path is shown in Figure 3. The upper part of Figure 3 is a rough representation of the path of the video signal, taking into account the large blocks, from the input from the camera to the Gigabit Ethernet output from the VVSP module. The lower part of Figure 3 presents in detail the architecture of the VVSP module with all the essential components used. Additionally, due to the complexity of the module, the upper and lower part of the image are interconnected by arrows, so that the reader can understand the flow of video signals in the presented architecture.

To enable communication with all components of the EO system, the VVSP must have appropriate interfaces integrated. The modularity of the VVSP module allows the interface to easily be changed and configured. The VVSP module allows the reception of images from cameras that use one of the following standard video interfaces: HD-SDI [23], CameraLink [24] and the analog composite PAL interface [25]. The maximum supported resolution format is 1920 × 1080 (FullHD) at 60 frames per second. The VVSP module can control all camera and lens parameters such as resolution, frame rate, gain, exposure, zoom and focus lens position, field of view adjustment (FOV), etc. The VVSP enables Pan/Tilt control, LRF, GPS, DMC, etc. The VVSP also has a special separate interface dedicated for communication with IMU sensors. As shown in Figure 3, the VVSP module consists of four separated PCBs: an interface board, an FPGA carrier board (FCB), a Jetson TX2 carrier board (JCB) and a board for Sensors, Signals and Control (SCC). The interconnection of these boards is achieved with high-quality board-to-board connectors that provide high data throughput in various ambient conditions.

The function of the interface board is to convert a video signal from one of the three supported camera interfaces (HD-SDI, CameraLink or PAL) to a parallel interface that is fed to the FPGA input. In order to achieve modularity, two variants of this type of board were designed. One type of this board, designated CLIf (short for CameraLink interface), with integrated SDR CameraLink connectors, allows one to receive video signals from two cameras that support the BASE CameraLink standard or from one camera that supports the MEDIUM CameraLink standard. The second type of interface board, designated as AnSDIf (derived from Analog and HD-SDI), has two integrated interfaces, one each for HD-SDI, and a composite analog PAL video interface. Both interfaces are connected via HD-BNC connectors.

The FPGA chip is integrated on the FCB board and performs video signal processing by introducing an extremely small delay in the video pipeline. The FPGA performs video signal conversion and preprocessing and can also apply some video algorithms that are suitable for this architecture, such as, e.g., pseudocoloring. The FPGA also performs color space conversion functionality in a very efficient way. A very important role played by the FPGA is the buffering of the video signal, which is discussed later, in a separate section. Another application of the advantages of FPGA technology is reflected in the precise synchronization of data from the gyroscope with the image, which is necessary for the IMU stabilization algorithm. In addition to the above applications, FPGA can also be used for the dynamic range extension algorithm shown in [26]. A stable video link is established between the FPGA and the application processor, so that this channel, after booting the VVSP module, sends either an image from the camera or a test pattern. In this way, the application processor is relieved of the worries about the camera at the input and the type of its video interface. It should also be noted that on the FCB board, there are HDMI interfaces with sending/receiving a raw signal to/from the outside world. The basic application of the FPGA on the VVSP platform is presented in [17].

The application module is integrated on the board with the abbreviated name JCB. This module is connected to the FPGA module via the previously mentioned link, and the video signal comes to it through the MIPI-CSI-2 interface [27]. As the application module is based on the Linux operating system, the corresponding driver presents this interface as a separate video device from the Linux device class [28]. After the described interpretation, the video signal will be further used as a source for video signal processing algorithms. In order to achieve the functionality of distributed data processing, the VVSP module on the JCB board has an integrated seven-port manageable Gigabit Ethernet switch. All devices with an Ethernet interface are connected to this switch, and mutual communication between VVSP modules is realized, as well as communication with the outside world. It should also be noted that a compressed video signal goes through this switch as a result of applying all processing on the VVSP module.

The board on which, in the VVSP stackup, most of the connectors with interfaces for connecting devices of the VMSIS3 system are integrated is SCC. This board has additional Ethernet connectors that are connected to the seven-port switch. Configurable serial interfaces are also integrated, including UART, RS232, RS422 and RS485. There are also GPIO signals, motor drivers for calibration cover for uncooled thermal cameras, relay switches, pressure, humidity and temperature sensors, etc.

### 2.2. Signal Processing

Video signal processing is a particularly very important aspect of VVSP module development and makes a significant contribution to intellectual property. It is especially important that signal processing is carried out in real time, with minimal delays and as few dropped frames as possible. In order to successfully realize this, as mentioned earlier, signal processing is performed on the FPGA module and on the application module. Both of these units are discussed here.

#### 2.2.1. Video Signal Processing and Frame Buffering on FPGA Module

**Figure 4:**
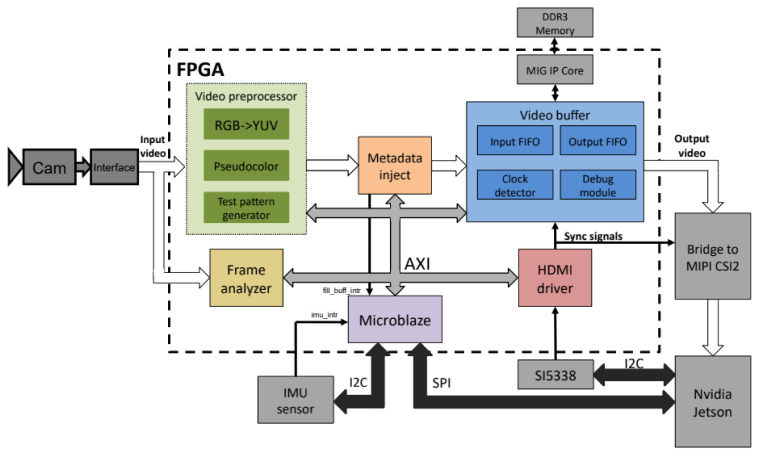
FPGA code architecture.

The complete organization of the block modules within the FPGA is shown in Figure 4. It is clear that there is an instance of the Microblaze microcontroller that is connected via the AXI bus to the following blocks: frame analyzer, video preprocessor, video buffer and HDMI driver. When a parallel video signal from the conversion chips on the interface board reaches the FPGA module, it carries the pixel data being transmitted and the corresponding sync signals. The frame analyzer only accepts synchronization signals, and on the basis of them, it calculates important image parameters, such as resolution and frame rate, and they are further retrieved by the Microblaze microcontroller.

The video signal from the input comes to a video preprocessor module consisting of a color space converter (RGB <-> YUV), a pseudocoloring module (which adds colors to the monochrome image in a user-defined way) and a test signal generator. Before the processed video signal from the preprocessor module reaches the video buffer, it passes through the inject metadata module, in which some metadata are entered, such as data from inertial sensors, frame counters, timestamps, etc. The video buffer provides synchronous writing and reading of data from DDR memory. Namely, a memory interface in the form of IP core is instantiated here using MIG IP components [29], whose role is to provide an interface to external DDR3 memory where entire frames will be stored. The video buffer also has two FIFO buffers: one that controls the process of the writing frame from the camera into memory and the other that controls reading frames from memory and forwarding them. An HDMI driver is a module that reads frames from a video buffer and forwards them to the FPGA output, over an appropriate MIPI–CSI-2 interface bridge to the application module. An example of the realization of connecting an FPGA to an application processor but via a PCI express bus is presented in [30].

The buffer involves the allocation of memory space, in this case DDR3 memory, sufficient to store an entire frame. The simplest, but also the worst way to buffer frames is to use single buffering. In that case, due to the inequality of the writing and reading clocks, the process of writing and reading is overtaken, which manifests itself as the appearance of a horizontal line on the displayed frame. The appearance of the line is a consequence of catching up with the process of writing and reading, so in one part, the reading process approaches the freshest frame, and in the other part it reads the data of the previously written frame. It is clear that this approach should be avoided.

The application module will have no problems with reception if the data coming from the FPGA goes over a stable link. If in some cases the camera clock is used for the reference frequency of this link, it will lead to jitter, which will confuse the driver on the side of the application module, since it is not resistant to this type of problem, and it will start rejecting frames or entering irregular condition. Therefore, a stable clock source must be used for the reference link frequency. However, even the slightest difference between the two clock sources, the one from the camera and the one that is the reference for the link, requires the frames to be buffered. This is something that needs special attention during development, as it can cause big problems later, so the method of buffering frames is described in detail.

The problem of the appearance of a horizontal line can be solved by double buffering. In this case, another buffer is allocated in memory. When double buffering is used, a new logic is introduced to control the change or repetition of the buffer over the write and read processes. The decision on whether the process will change the buffer or repeat the same buffer is made at the time the last pixel in the current buffer is processed. At that moment, how far the indicators related to both processes are from each other is considered, and on the basis of previously determined limit values that are entered in the appropriate registers, a decision is made. The problem with double buffering is the frequent repetition of the buffer, which manifests itself as image freezing. Namely, it is a limit moment for certain clock values when they vary slightly in one direction and in the other. They need some time to disperse a bit, but by then, the frames will be frozen. The described problem is also solved by introducing an additional buffer.

**Figure 5:**
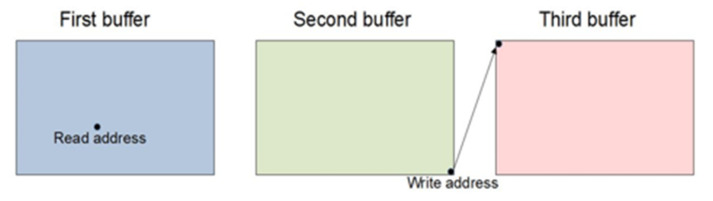
Triple buffering logic: buffer change for write process.

By introducing an additional buffer, the triple buffering of frames is performed. The read process is in one buffer, the write process is in another, and there is always one free buffer. Again, in this case, there should be logic to change the buffer. The logic for switching between buffers in the enrollment process is to always switch to a free buffer. An illustration of this logic is shown in Figure 5. An example of triple-frame buffer FPGA implementation is shown in [31].

**Figure 6:**
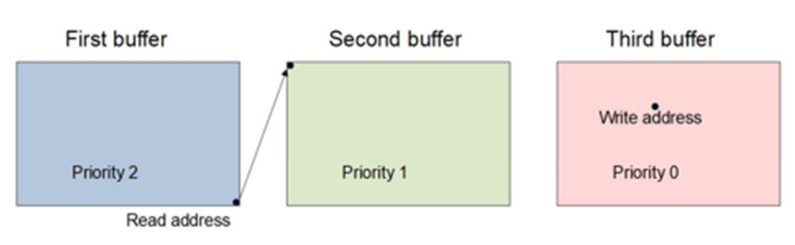
Buffer change for read process in triple buffering.

The logic in the read process to change the buffer must follow where the last frame was written, assigning priorities to the buffers when reading the last pixel in the current buffer. The highest priority is assigned to the most recently entered buffer (marked with priority 0), while the lowest priority is assigned to the oldest buffer (marked with priority 2). The logic is realized so that the reading process always passes into the buffer marked with priority 1. This is shown in Figure 6. Within the described realization, it is possible to repeat the buffer if the read process is faster than the write process. Additionally, if the write process is faster than the read process, then there will be a frame skip. In this way, it is ensured that the processes do not catch up with each other and that the frame does not freeze. The main disadvantage of this approach is the increase in delays.

**Figure 7:**
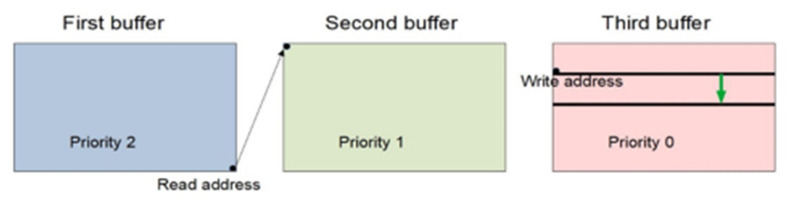
Limit values for buffer change in modified triple buffering.

Latency reduction can be achieved by allowing the read process to enter the buffer in which the write process currently is. An important condition is that the writing process has gone far enough, so it has exceeded a certain limit value. This buffering procedure is referred to as modified triple buffering. The problem that arises here is the fact that the clocks of the write and read processes vary over time and seem to catch up with each other. This is the reason for a large number of skips or repetitions of a significant number of frames. At that point, the write process pointer is close to the limit value that determines whether the read process can enter the same buffer or repeat the read buffer. This behavior will continue until the pointer of the write process is far enough away from the limit value. In order to prevent the occurrence of the described phenomenon, hysteresis with two given limit values is introduced. The write process checks when changing the buffer whether the write process pointer has reached the first limit value. If this condition is met, another limit value is set, which defines hysteresis. In this way, the decision to change the buffer for the read process is made more safely, and the example of the boundary heather is shown in Figure 7, where the set crossing zones are marked in the third buffer, and the green arrow illustrates the movement of the limits. When the write process pointer reaches the second limit value, the change will occur again, but the first limit value will be set at that point (the green arrow will have the opposite direction). In this way, the delay is significantly reduced.

**Figure 8:**
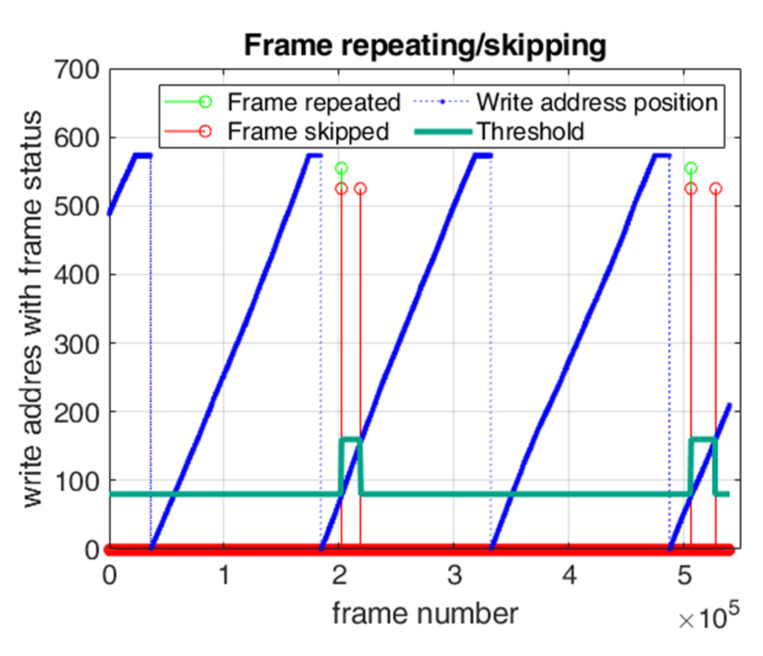
Modified triple buffering: hysteresis change.

In Figure 8, the behavior of the system when modified triple buffering with hysteresis changes is applied is shown. With blue dots, the pointer for the write address is shown, and it travels from address zero to maximum address defined by the height of the picture that the camera gives on its output, which is to be transferred. When it reaches the maximum address, it stays for a length of time to wait for the next picture datum to arrive, since the frame is slightly larger than the useful part of the picture. Repeated frames are marked with a light green line, while skipped frames are marked with a red line, and the hysteresis limit is marked with a dark green line. When a change to the hysteresis limit occurs, the repetition of frames can happen as well as skipping of frames or both situations. The repetition and skipping of frames can only occur when hysteresis limits are changed, and it happens once in every 250,000 frames. In Table 1, the behavior of the system and the latency of transferred picture data in cases of single buffering, double buffering, triple buffering and modified triple buffering are shown.

#### 2.2.2. Algorithms Application—Video Signal Processing on Application Module

Complete video signal processing on the application processor is designed to apply algorithms to the video signal received from the input Linux video device (e.g., dev/video, which receives content from the FPGA), and the result is a compressed video signal with applied algorithms that will be streamed via Ethernet. In order to realize this idea, the well-known GStreamer library [32] is used. GStreamer is a framework written in the C programming language and uses the concepts of object-oriented languages, as it is based on GObject and GLib libraries. GStreamer uses basic elements that communicate with each other via “pads”. The set of interconnected elements where data travels from the “source” element pad (the producer side), to the “sink” element pad (the consumer side), and performs various tasks along the way, is called a “pipeline”. Encapsulating one or more elements in a form of plugin allows GStreamer to use them. When used in the Linux operating system, the plugin is basically a loadable block of code in the form of a shared object file (.so).

**Figure 9:**
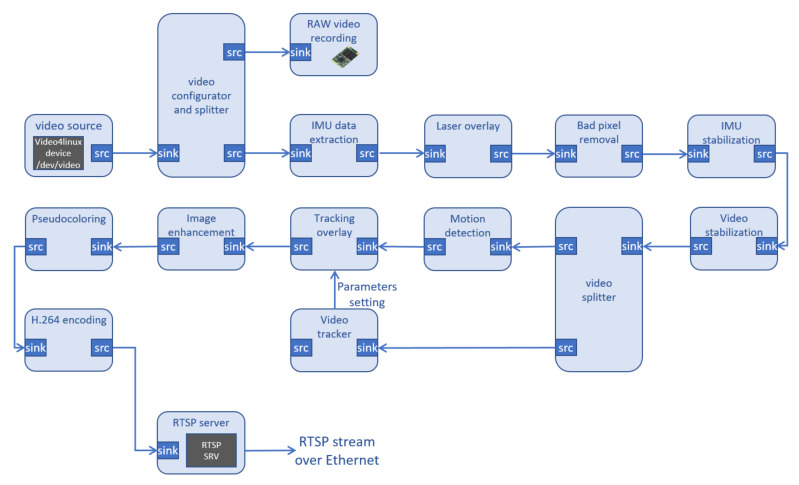
VVSP video processing pipeline.

The realization of the pipeline for the algorithms’ application on the VVSP module is shown in Figure 9. In this particular example, GStreamer elements may have certain functionalities, such as configuration, splitting or text overlay via video signal, etc. Each algorithm that processes video signals in any way is implemented as a separate plugin. Additionally, each of the algorithms in the pipeline can be turned on or off depending on the need for its functionality.

Gstreamer elements can have certain functionalities, such as a video signal configurator and splitter or an overlay layer. Additionally, they can be implemented as plugins that represent specific applied algorithms, including: bad pixel removal [33], video stabilization, IMU stabilization [10], object tracking [11], video enhancement [12], pseudocoloring, motion detection, h.264/265 encoding (with a dedicated hardware co-processor), etc. All these algorithms are developed in Vlatacom Institute. Finally, a video signal is prepared for streaming by instancing the Real Time Streaming Protocol (RTSP) Server [34].

### 2.3. Software Architecture

**Figure 10:**
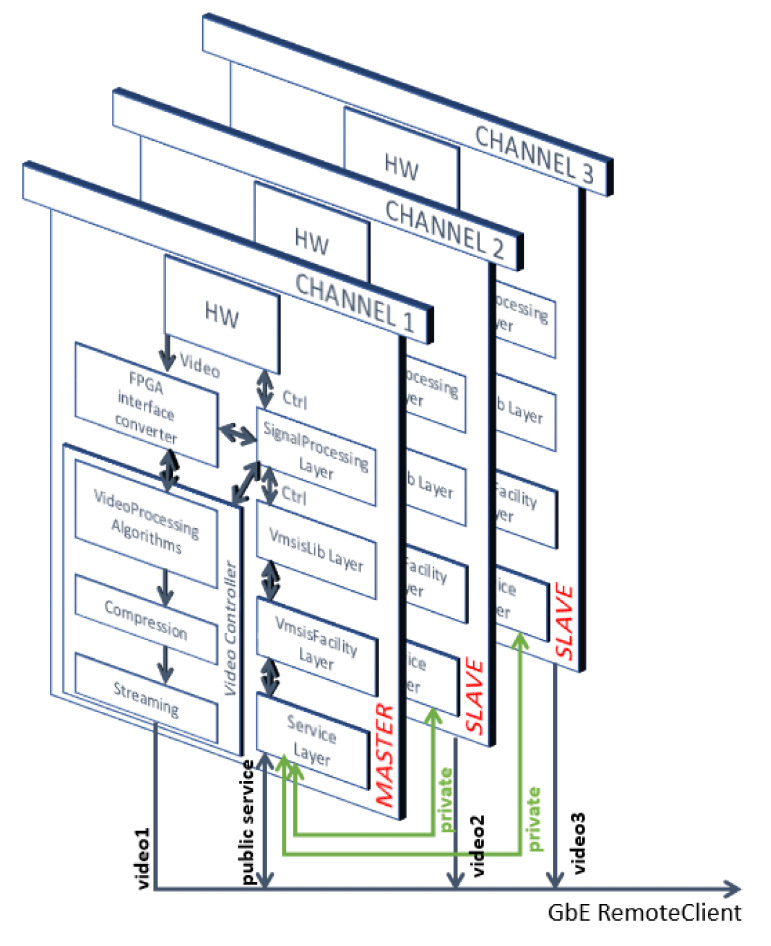
VVSP software architecture.

Another aspect of the development of the VVSP module is the software design, which was carried out completely at the Vlatacom Institute, with basic architecture shown in Figure 10. From the software side, the VMSIS3 system is a standalone, electronic device that produces imagery data using one or more image sensors and optical devices in different electromagnetic spectrum ranges and provides these data in the form of a video stream to end-user devices or applications. The VMSIS3 also provides control and monitoring of various operational parameters of the system. If we consider the VMSIS3 system from the communication side, it is in fact an Ethernet network device that is controlled via a single IP address, which sends a video stream via multiple IP addresses. These IP addresses are used by end-user devices and applications, designated as remote clients, to accept the provided video stream, as well as to access the public services of the VMSIS3 system.

A typical configuration of the EO system VMSIS3 consists of two or three image sensors, coupled with appropriate optical lenses, all mounted on a moving platform (pan-tilt), together with the EO System Controller. The term EO System Controller designates a set of computation processors with associated hardware, that present VVSP modules, and software components, responsible for EO system control, image acquisition and processing, as well as video streaming. The EO System Controller responsibilities are split between separate VVSP modules running the controller’s software components.

The term EO Channel refers to one coherent group of devices and software components gathered around one image sensor or one video stream. EO Channels may share some of the devices, such as the pan-tilt, for example. Each EO Channel has at least one dedicated VVSP module for control and video processing on that EO Channel. VVSP modules with this role will be referred to as Channel modules. An additional role that the VVSP may have is to provide a public interface for the EO System and to communicate with internal channel modules, which is denoted as master controller. Regardless of its role in the EO system, the same VVSP software runs on all VVSP modules, and its role depends on its configuration.

VVSP software runs in a single operating system process and can have a master controller, slave role or both. It should be noted that there are two purposes of VVSP software related to the EO channel:

- The first one is related to the acquisition, compression, processing and streaming of the video frame on the public side of the EO system;
- The second one is related to device control (camera, camera lens, tilt, etc.).

The master controller enables communication with remote clients using appropriate services. The VMSIS3 within the VVSP module relies on the use of web services implemented in a subset of the ONVIF Profile S protocol [35] with some custom extensions.

VVSP software is a very complex solution organized in several separate C++ projects that are grouped into several layers. The affiliation of a layer depends on the level of abstraction to which it belongs, with the highest layer (top) being the closest to the remote client, while the lowest layer (bottom) is closest to the hardware. The bottom-to-top layers are organized as follows: the Vmsis signal processing layer, Vmsis Lib layer, Vmsis Facility Layer and Vmsis Service Layer. From an integration point of view, these projects are presented as a group of C++ library projects that are linked into an executive application project.

Each device in the VMSIS3 system is supported in the Vmsis signal processing layer at the register access level, which means that for each device, there is an interface converter from the device’s native communication protocol to a GenTL register-based access interface [36]. The GenTL interface introduces an idea of presenting any device as a set of registers on different addresses, which accept data of specific types. Each feature of the device is related to a register. GenTL is based on GenICam [37]. The GenICam standard provides a generic programming interface for all kinds of devices, no matter what interface technology they use. The Vmsis Lib Software Layer represents any device or software component in the VMIS3 system as a generic device. According to the GenICam specification, it applies the level of nodes, devices, interfaces and systems. The Vmsis Facility Layer refers to the identification of devices by classifying them into categories. A generic device from the Vmsis lib layer in the Vmsis facility layer becomes a camera device with a lens with its specific characteristics, a Pan-Tilt device or a laser rangefinder, etc. The top abstraction of this software layer is a facility. A facility represents some subsystem or group of functions provided by the EO system, so there are following facilities in VSP software: a core facility, media facility, Ptz facility, imaging facility, component facility, Lrf facility, maintenance facility and video facility. The whole Vmsis facility layer resides in the Vmsis facility library. The Vmsis Service Layer implements ONVIF Profile S protocol services, as well as custom services according to the needs of clients and systems in which the VMSIS3 is integrated. The VVSP executable is run using the Linux operating system service manager.

### 2.4. Network Architecture

**Figure 11:**
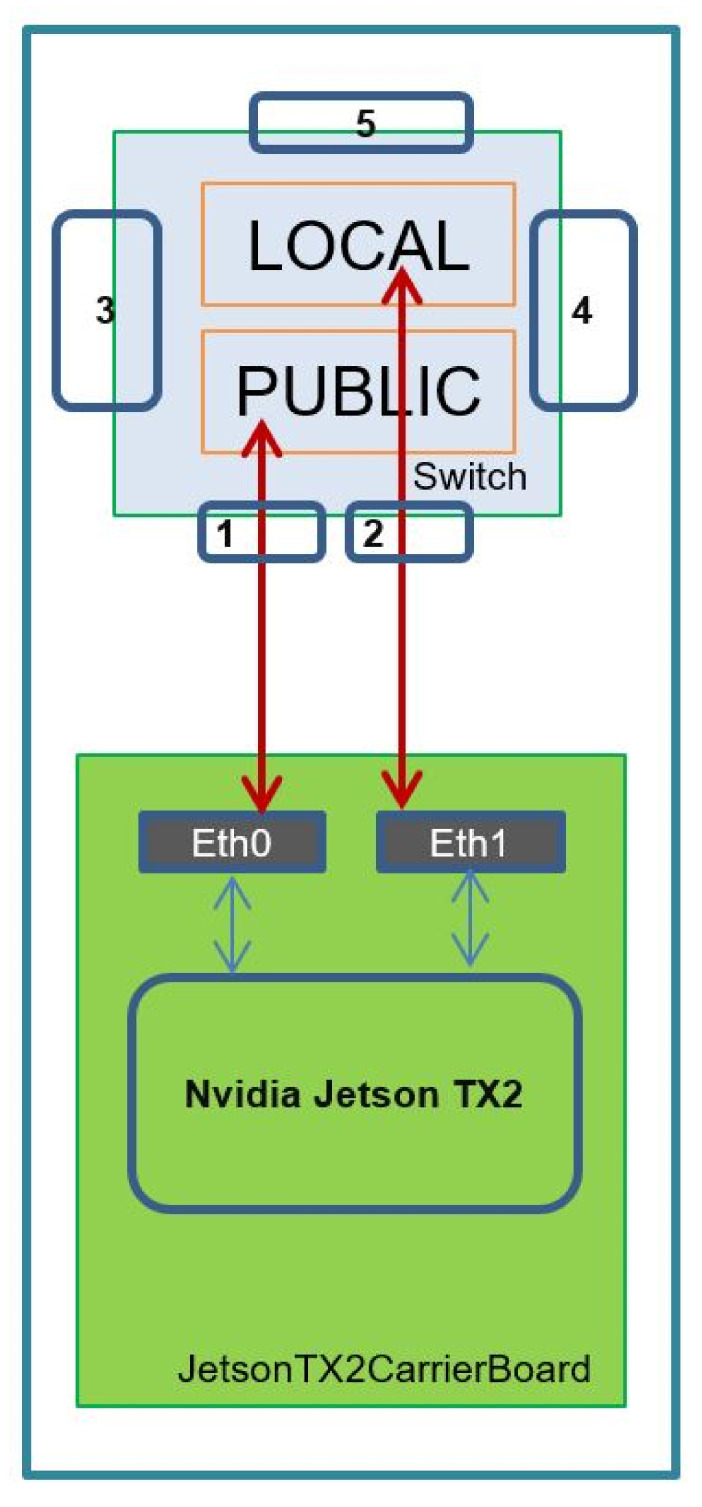
VVSP module networks.

In order to fully enable the distributed architecture applied in the VMSIS3 system, special attention is paid to the network architecture when designing the VVSP module. On the application processor, two network cards (NIC) are integrated within the VVSP module, so that the processor has two access ports to its integrated managed Ethernet switch. This implementation allows each VVSP module to have two separate networks, private and public, as shown in Figure 11, in order to improve the security of the entire system.

**Figure 12:**
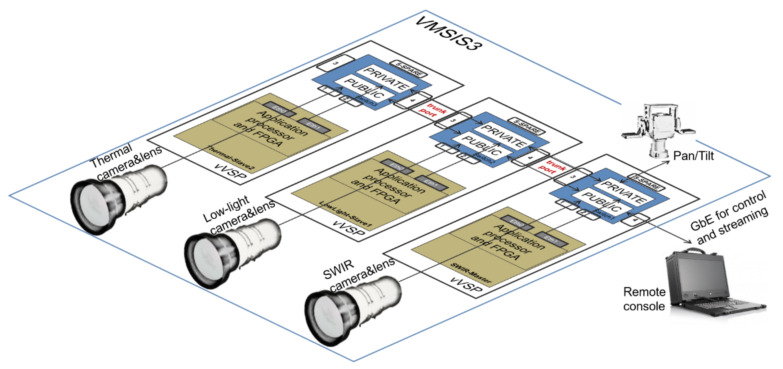
VVSP network topology.

VLAN technology [38] is used to enable the existence of two (or more) isolated networks. The point is that VLAN tags are applied to Ethernet packets, and by properly recognizing these tags, it is possible to create the functionality of network traffic that is physically on one network but acts as if it is shared between separate networks. It is clear that in this way, the traffic in one network is separated so that the physical LAN network is divided into several domains connected in a logical sense. Thus, the private and public part of the network on the VVSP module are separate VLANs, and together, they are transmitted through the TRUNK port, which can carry both of them. The application processor accesses the public part of the network via the Eth0 interface, while the private part of the network is accessed via the Eth1 interface. The topology of the VMSIS3 network is presented in Figure 12.

The figure shows a system consisting of three channels, and thus three cascade-connected VVSP modules. The first VVSP module in the cascade is assigned the role of master controller, and one of its switch ports is configured for public access to the external network. This port provides access to part of the public network of the VMSIS3 system, and thus to its service. Additionally, all compressed streams generated from the RTSP server on Channel VVSP modules are transmitted through the public part of the system and are accessible to the outside world (remote devices and applications). All communication that is protected from the outside world is done in a private part of the network. Additionally, all devices that should be visible only within the VMSIS3 system are connected to this part of the network. These can be cameras that have a network interface for configuration or, as shown in Figure 12, a pan-tilt device that has a native network interface. If at any time it is necessary to access network devices from a private network from the outside world, this can be done by appropriate port forwarding. This is, of course, a strictly controlled operation and requires multi-level access protection.

### 2.5. Integration

**Figure 13:**
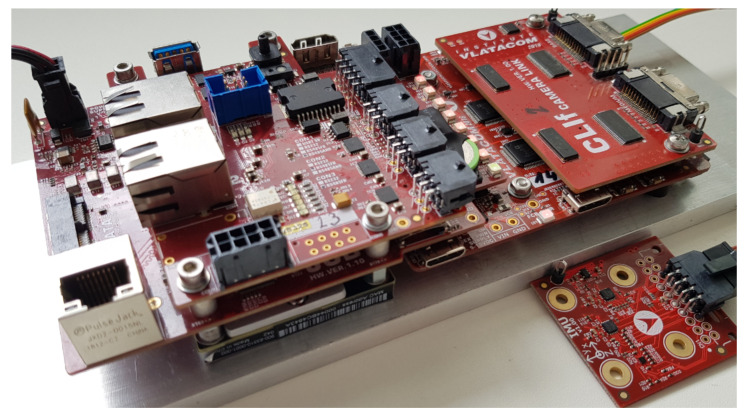
VVSP module with connected IMU sensor.

A fully assembled VVSP module with all stacked boards including the interface board (CLIf in this case) and connected IMU sensor is shown in Figure 13. The stacked board structure combined with high integrated technology with reliable board to board connectors allow miniature module dimensions, so the complete VVSP module has the size of an open hand. The power consumption of a single module is about 10–15 W. The VVSP module is intended to be packed in the same housing as the camera, lens and other related equipment, and wiring connections between the VVSP and the mentioned equipment are all realized inside the housing with no external cables, except for the main power supply and communication link of the VMSIS3 system, which passes through the pan-tilt slip ring, so that the system movement of the N × 360° is enabled.

The IMU sensor shown in Figure 13 is intended to be mechanically coupled with the camera sensor, so the IMU stabilization algorithm has the best possible performance. The VVSP module is connected with IMU sensor via a dedicated interface.

**Figure 14:**
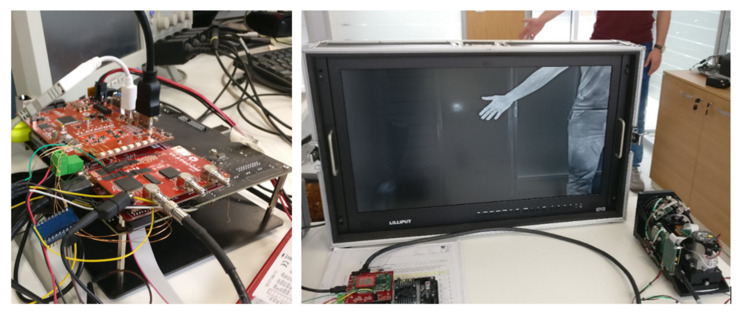
VVSP module during development stage.

In Figure 14, parts of the VVSP module are shown during the development stage. On the left image, a stack of PCB boards, which includes the first revisions of JCB and FCB boards with AnSDif interface board, IMU sensor board and wires, is connected to an oscilloscope for testing and debugging. At this stage of project development, testing of video and communication interfaces is done at the hardware level. On the right image, testing of a part of a video pipeline via the FPGA processor before it comes to the main application processor for further processing is performed. A video stream is gained with signal acquisition and FPGA preprocessing from thermal camera and is shown on the output monitor.

**Figure 15:**
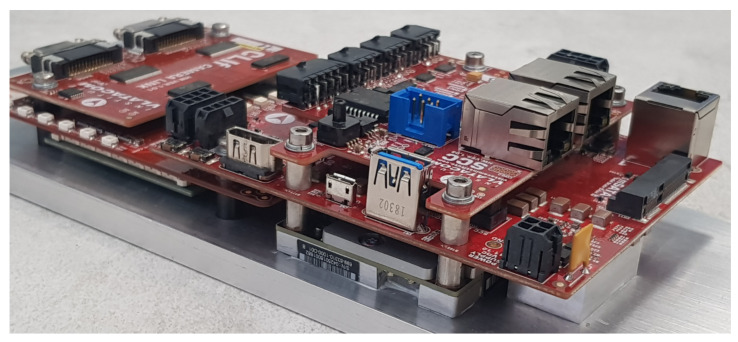
VVSP fanless solution.

**Figure 16:**
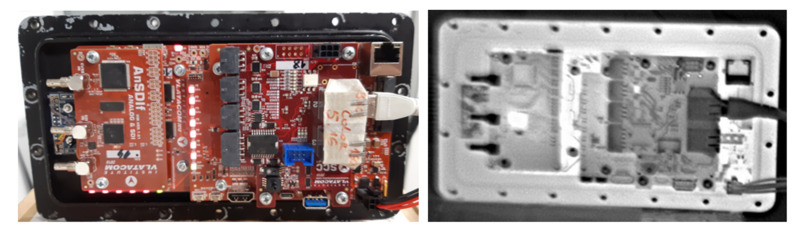
VVSP module top view filmed with lowlight and thermal camera.

One of the great advantages of the VVSP module that should be mentioned is the operation in a fanless mode. An aluminum support, to which critical components regarding heat dissipation (Jetson TX2 and FPGA module) are directly connected, is placed on the wall of VMSIS3 housing. This wall is ribbed on the outer side, so that the heat can directly be transferred to the environment, which is the most efficient method of passive cooling. A thermally conductive material is placed between the module and the carrier. The solution for passive cooling of the system with aluminum support is shown in Figure 15. A view of a VVSP module in the fully operational stage filmed with lowlight and a thermal camera filmed with the VMSIS3 system is shown in Figure 16. The completed VVSP module with FullHD resolution at 30 frames per second, which is prepared to be installed in VMSIS3 housing, is tested before the installation. The VVSP shown in Figure 16 streams a test pattern in FullHD @ 30fps, and on right side of the image, the same module is filmed with a thermal camera so that distribution of temperature along entire stack of boards can be visualized. Gradation for thermal coloring in this case goes from black (cold) to white (hot).

**Figure 17:**
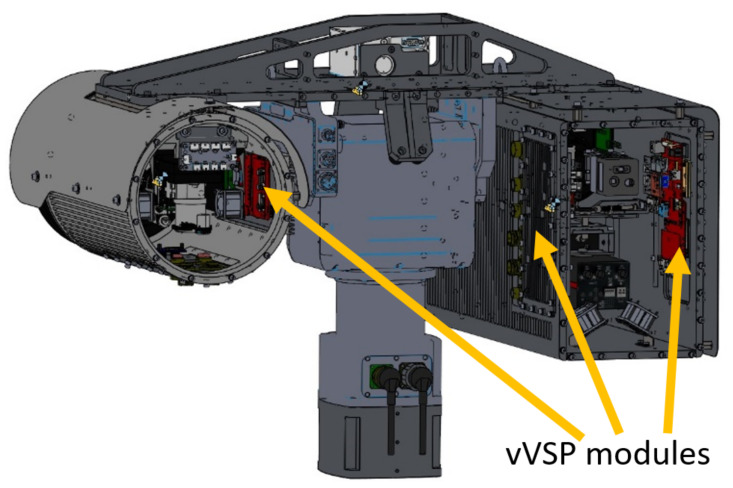
Positioning of VVSP modules in VMSIS3 system.

The usage of the implemented passive cooling allows smooth operation of any VMSIS3 system in the temperature range from −25 °C to +55 °C of ambient temperature. Once installed in the VMSIS3 device channel, the VVSP module becomes hermetically sealed, since it is of great importance to stop the penetration of moisture inside the channel to prevent any physical damage that moisture can cause to electronic components. An example of an integrated VVSP module connected with other equipment in the system is shown in Figure 17. This example shows the complexity of the arrangement of components within the EO system and how little space is set aside for the VVSP module. This is precisely the reason why the miniaturization of the video signal processing module was necessary. As a result, the VVSP module can be used not only in stationary applications, but it can also be integrated in vehicles where it is mounted on a pull-out pillar. An example of positioning all VVSP modules with passive cooling solution in a VMSIS3 system with T-shape pan-tilt is shown in Figure 17.

## 3. Results

In this section, certain results and comparisons regarding performances of video processing algorithms implemented in the VVSP module’s software architecture are presented.

The implemented algorithms are taken from the references [10,11], in which one can find more details about implementation and performance, which are beyond the scope of this paper. The goal of this experiment is just to give a brief insight about the performance of the VVSP rather than to compare it with other solutions. Please note that due to the VVSP concept, there is a plenty of room for algorithm implementation optimization by distributing processing tasks between the FPGA, multicore microprocessor and GPU. This optimization is beyond the scope of this paper.

**Figure 18:**
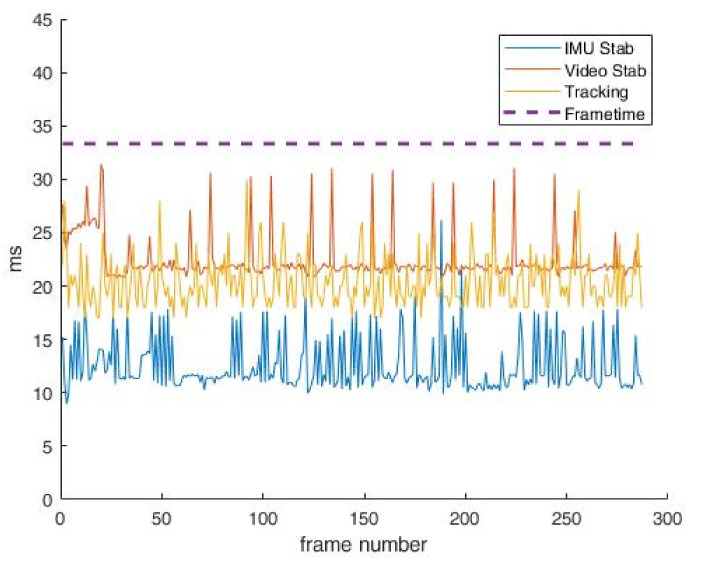
Algorithms’ execution times at video stream of 30 fps in FullHD.

In Figure 18, the time of execution of all applied video processing algorithms, including IMU stabilization, video stabilization and tracking with streaming a FullHD video of 30 frames per second is shown. The mentioned resolution is used in this test since it is the highest possible resolution that the VVSP module currently supports. X axes represents the number of frames, while Y axes represents the time needed for the execution of every applied algorithm along with time needed for one frame. It is clear that every frame in this case will be transferred, since every applied algorithm can finish its calculations in time before the next frame comes to signal processing.

**Figure 19:**
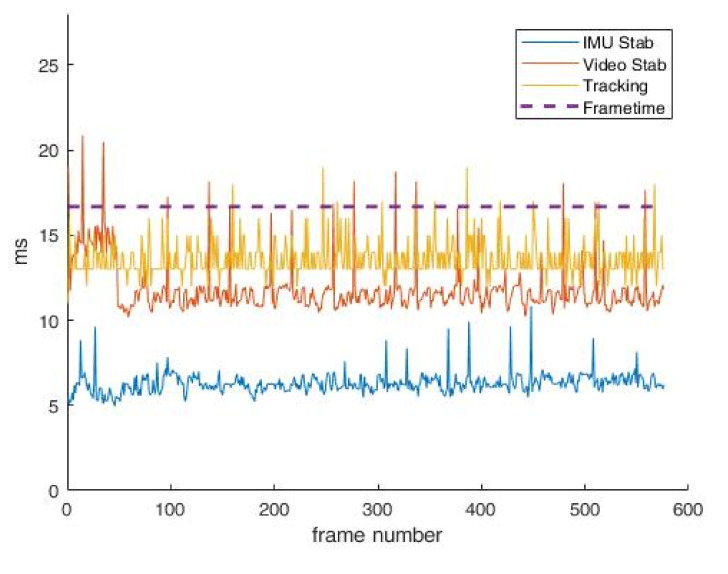
Algorithms’ execution times at video stream of 60 fps in VGA.

Since testing for algorithms’ execution time when the frame rate is 30 frames per second shows perfect results with no skipping of frames, the second test is performed with video streaming when the frame rate is 60 frames per second. In Figure 19, the time of execution of every applied algorithm when streaming a VGA-resolution video of 60 frames per second is shown. It is shown that not every frame in this case will be transferred, since video stabilization and tracking algorithms cannot always finish their calculations in time before the next frame comes to signal processing. In this case, the percentage of dropped frames is about 2–3%, as shown on Figure 19, which is irrelevant for the human eye.

The VVSP module is subjected to testing in different temperature ranges via a programmable heat air circulation environmental chamber. The goal of this experiment is to test the functionality of the developed device in temperature range from −20 °C to +55 °C and to check the behavior of the system in extreme temperatures. The time of exposure of the VVSP module to extreme temperatures is from 25 to 40 min. By exposing our hardware to the temperature test, special attention is paid to temperature changes from −2 °C to 2 °C, since in this temperature zone, condensation can be a huge factor that can possibly damage the hardware, so that the transient is programmed to last longer. Heatsink and ambient temperature measurement is achieved with contact temperature sensors, while the temperature measurement of the FPGA module and NVIDIA Jetson TX2 is performed using their internal temperature sensors. Samples from each sensor are acquired by specially designed application to accept data from each sensor every 60 s and recorded.

**Figure 20:**
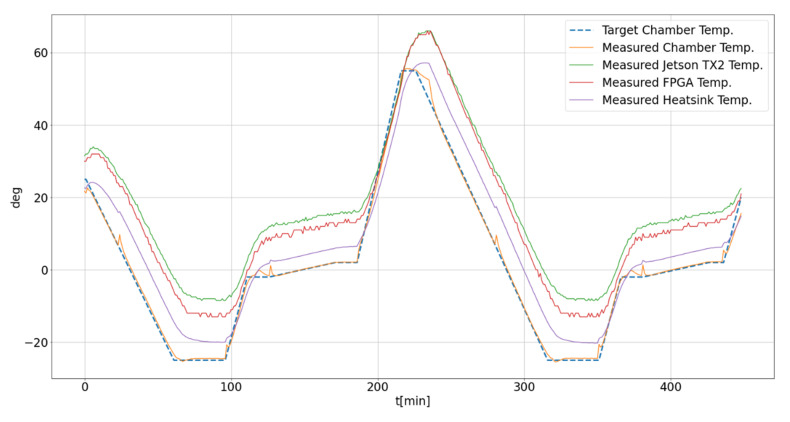
VVSP module temperature endurance test.

The chart in Figure 20 shows the results of temperature profile testing in a programmable air chamber with Y-axes showing the temperature (in Celsius degree) and the X-axes showing the time of exposure to certain temperature (in minutes). The orange line represents the ambient temperature, the blue dotted one is the programmed target temperature of the air chamber, the green line is for the Jetson TX2 module that streams video in FullHD resolution, the red one is the temperature of the FPGA module and the purple line represents the temperature of aluminum support for passive cooling. All these results are expected, and none of the tested modules show any degradation of video stream or algorithms’ execution during the temperature test. The temperatures of all critical components follow the ambient temperature and are slightly heated above it. FullHD resolution with 30 frames per second is chosen for the temperature stress test, since that is the highest possible resolution that the VVSP can provide and requires more processing and memory resources to be used than the other supported resolutions.

## 4. Discussion

The main goal when the project started was to develop a unique video signal processing platform that can accept video input from any camera with a standard industrial interface to process the signal and output it via IP. Special attention was directed to the size, weight and performance of such a platform. All of the primary goals were achieved by developing the VVSP, a platform that can easily be installed alongside a camera in housing of a VMSIS3 system. In this paper, the entire workflow of the project was described in detail, with special attention to hardware architecture, which includes designing complicated PCB boards as carrier boards for signal processing components, algorithms’ development and their application on those components, entire signal flow, software organization in the main application processor, network topology and the integration of the developed platform into a complete enclosed system for usage in border control and military surveillance applications. There were a lot of challenges to overcome during the development stage of the project. One of the most difficult and time consuming was the implementation of triple buffering and memory manipulation of the FPGA processing module as well as developing and integrating video processing algorithms on the main application processor and organizing entire software on it, since most of the sensors and actuators besides video are controlled by the main processor.

VMSIS3 systems with VVSP modules have more than 3 years of successful work on the field in various ambient conditions. The systems are employed in border control and field surveillance applications in desert, maritime and continental climate weather conditions as stationary units as well as mobile units mounted on the armored vehicles. Systems did not show any degradation of performance in the ambient temperature range from −25°C to +55°C [39]. Based on this experience, we summarized the VVSP concept contribution described, which is given in Table 2.

**Figure 21:**
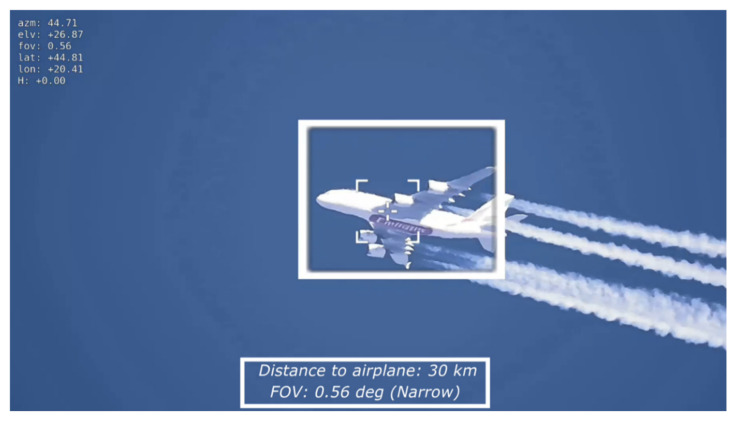
Long-distance target tracking.

During the testing phase of the VVSP module employed in the VMSIS3 system, long-distance target tracking was tested from a rooftop of our Institute. Figure 21 shows the tracking algorithm performance when the commercial aircraft was spotted at a distance of 30 km, and it was successfully tracked.

Considering the fact that development of the VVSP module started in 2017, most of the components used for the realization of the project were and still are very reliable and well represented on the electronics market. The most critical component is the main processor Jetson TX2, since its lifecycle is predicted to end in January 2025 [40]. During this project, a lot of knowledge regarding FPGA technology and the Linux operating system when employing GPU cores was adopted, so migration to newer and more powerful signal processing modules could be done with slight modifications of an existing system. One of the possible reasons to migrate to newer design, besides more processing power, is even more miniaturization that could now be achieved by using System on Chip with an FPGA and the main application processor packed in the same chip. Additionally, rapidly evolving algorithms of artificial intelligence could be implemented on the VVSP module as one of the future upgrades of the system, but that would also demand an upgrade to a hardware platform that has more processing resources than Jetson TX2. The described VVSP platform has more general application than that of long-range surveillance systems. Virtually any multi-sensor system can utilize this platform, especially in sensor fusion applications such as the integration of radars and multiple cameras for target tracking [41]. Similarly, autonomous vehicle applications like those presented in [42] can utilize the VVSP platform without significant modifications.

## 5. Conclusions

In this paper, a complete hardware and software solution for capturing and processing image and sensor data specially tailored for long-range surveillance applications was presented. The developed platform can also be used for a various number of applications beside its main role. The greatest benefit of the described module is the distributed software, hardware and network architecture which makes the system completely modular and adoptable to accept any kind of sensor with slight modifications in either hardware, software or both. Additionally, one of the key benefits of the described module is its size, which makes the hardware structure easy to pack in various types of housings and can be used as a general platform for signal processing in different scenarios and applications.

A topic of future work includes the further development and implementation of artificial intelligence algorithms and more hardware and software modifications to make the entire system even smaller in size and more powerful with regard to its processing resources.

## Figures and Tables

**Table 1:** Buffering techniques.

Buffering Technique	Latency	Behavior
Single buffering	Part of frame	Low latency/sync problem
Double buffering	0 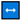 1 frame	Low latency/sync problem
Triple buffering	1 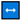 2 frames	Higher latency/no sync problem
Modified triple buffering	Limit 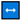 1 frame + limit	Medium latency/no sync problem

**Table 2:** VVSP concept contribution vs. traditional concept approach.

Task	Traditional Approach	VVSP Concept	Contribution
Video signal monitoring	Analogue PAL or NTSC digital (HD) SDI or CoaxPress.	Digitalized H264.	Easier interfacing to command control systems. No need for additional hardware/software.
Camera interfacing	PAL/NTSC—easy (HD)SDI/CoaxPress—easy, limited range CameraLink or LVDS—difficult, converter to HD-SDI needed introduced latency and possible format change.	Easy connection to any interface on camera with just choosing proper interface board. This is important for thermal and SWIR cameras, which predominantly utilize CameraLink or LVDS interface for maximal performance.	Interfacing to any camera interface is direct without any resolution change or additional latency.
Pan-tilt control	Via Pelco-D, ONVIF or similar protocol.	Via ONVIF protocol. For a critical algorithm such as target tracking, pan-tilt drive is direct from the VVSP without any additional latency.	VVSP has an advantage in applications executed directly on HW- and AI-based control.
Pan-tilt positioner slip ring communication	Limited to coaxial or Ethernet interfaces; otherwise, converters are needed. Additional interface is needed for pan-tilt, LRF and lenses control.	Only uses one Ethernet interface for both video streaming, camera, lenses, LRF and pan tilt control.	Reduces requirements for pan-tilt positioner slip ring.
Fanless operation	Some processor modules may require additional cooling.	VVSP is completely passively cooled.	Efficient system cooling which is tested even in desert conditions.
Resolution	Limited to PAL or NTSC. High-resolution/dynamic-range imaging not supported. Digital interfaces have no limitations.	Full HD resolution and high dynamic range that is critical for, e.g., thermal imaging, which is fully supported.	Much better image quality.
Latency	Minimal latency before external compressing hardware is used.	About 300 ms. Please note that critical algorithms (e.g., tracking) are executed on vVSP, without any latency.	If an MSEOS is integrated into the C2 system, there are no differences.
Target tracking	Executes on external hardware that controls pan-tilt. For good performance, the controller should be installed close to the MSEOS.	Executes on VVSP. Utilizes full resolution and frame rate of all sensors. No additional latency. Very compact solution.	Considerable advantage for VVSP due to direct sensor access.
Image stabilization	Limited to image-based algorithms. Depends on the particular scene.	Can utilize different methods (e.g., IMU stabilization [10]).	Much more flexibility than the traditional approach.
Image fusion from multiple cameras (e.g., thermal and visible)	External hardware. Time synchronization and image registration might be difficult to calibrate.	A dedicated VVSP module can run image fusion algorithm acting as a “virtual video channel”.	More flexibility and easier calibration.
AI target recognition	Need to digitize the image prior to the application of AI modules. The concept limits resolution, which reflects on AI-based solution performance.	Full resolution of the image can be utilized for AI-based target detection application. In cases of a lack of computational power, additional VVSP modules can be added.	Much more flexibility, especially if target detection is linked to target tracking with pan-tilt movement in order to keep a tracked target in the center of the scene.
Image enhancement	No possibilities.	Easy implementation of various image enhancement algorithms.	Especially important for thermal imaging.
Laser range finder (LRF) application	External command for measurements. Targeting reticle is very hard to implement in continuous zoom cameras.	Simple implementation of targeting reticle even in continuous zoom systems [6].	Easier integration. Additionally, it enables the implementation of complex applications (e.g., LRF-aided target-in-lock indication for tracking systems).
Overall moving payload weight	Minimal or no extra payload.	Each VVSP module adds about 0.45 kg in weight.	This is a small drawback since the weight of cameras and lenses in long-range MSEOS is much bigger.
Private network for Ethernet devices	Additional manageable GbE switch is required.	A manageable GbE switch is integrated in the VVSP module.	VLANs configuration enabled. Important devices connected to the network can be hidden from the outside world.
Command–control (C2) system interfacing	Requires protocol conversion box for video digitizing and range extension.	Everything needed for C2 system integration is obtained via a single Ethernet port.	Easier interfacing to the C2 system.
System scalability (adding a new camera to the system)	System is limited to a certain number of cameras or requires additional hardware and/or system architecture redesign.	Only an additional VVSP is required. This module is connected to the existing system network over GbE.	This is made possible by the initial concept of distributed system architecture.
